# LH/FSH Ratio Is Associated With Visceral Adipose Dysfunction in Chinese Women Older Than 55

**DOI:** 10.3389/fendo.2018.00419

**Published:** 2018-08-02

**Authors:** Li Zhao, Chunfang Zhu, Yi Chen, Chi Chen, Jing Cheng, Fangzhen Xia, Ningjian Wang, Yingli Lu

**Affiliations:** Institute and Department of Endocrinology and Metabolism, Shanghai Ninth People's Hospital, Shanghai JiaoTong University School of Medicine, Shanghai, China

**Keywords:** LH/FSH ratio, obesity, visceral fat dysfunction, women, lipid metabolism

## Abstract

No study examined the association of luteinizing hormone to follicular stimulating hormone (LH/FSH) ratio with both visceral obesity outside the context of polycystic ovary syndrome. Thus, we hypothesized that the LH/FSH ratio was associated with visceral adipose accumulation and dysfunction among Chinese women older than 55. From 2014 to 2015, a total of 2,525 women aged 55–89 years were identified from a cross-sectional survey on the prevalence of metabolic diseases and risk factors in East China. Anthropometric indices, biochemical parameters, sex hormones and clinical characteristics were measured. Visceral adipose accumulation and function were identified by visceral adiposity index (VAI), Chinese visceral adiposity index (CVAI) and lipid accumulation product (LAP). Linear regression and logistic regression analyses were conducted to explore the association. A total of 1,462 (57.9%) participants had visceral obesity. In the linear regression, after full adjustment for demographic variables, metabolic factors, total testosterone (T), and estradiol (E_2_), LH/FSH ratio was positively associated with all indices estimating visceral obesity [B (95% CI): Log VAI 0.060 (0.030–0.090), Log CVAI 0.045 (0.029–0.061), Log LAP 0.103 (0.063–0.142), all *P* < 0.001]. Logistic regression analyses showed that the risk of visceral obesity increased with increasing LH/FSH ratio after controlling for age and smoking [OR (95% CI): 1.99 (1.52, 2.59), *P* < 0.001]. After further controlling for metabolic factors, the association was attenuated but remained significant [OR (95% CI): 1.89 (1.42, 2.53), *P* < 0.001]. The OR of visceral obesity in the fully adjusted model was 1.83 (95% CI 1.37, 2.45) (*P* < 0.001). Thus, high LH/FSH ratio was significantly associated with visceral adipose over-accumulation and dysfunction in women over 55 years old. This ratio may be an early marker for metabolic disorders in Chinese women older than 55, which warrants further investigation.

## Introduction

Visceral obesity reflects not only adipose tissue storage dysfunction but also triglyceride accumulation in ectopic sites including skeletal muscle, liver and pancreatic β cells (1). Compared with overall obesity, visceral obesity is more closely associated with metabolic syndrome (2), non-alcoholic fatty liver disease (NAFLD) (3), diabetes, and cardiovascular diseases (4). Traditionally, waist circumference (WC) and waist-to-hip ratio (WHR) were most widely used to estimate visceral obesity; however, they could neither distinguish between visceral and subcutaneous fat (5) nor precisely predict visceral adipose dysfunction. Thus, the visceral adiposity index (VAI) and the lipid accumulation product (LAP) were introduced (6). VAI is a new index for the assessment of adipose distribution and function that has shown promising capability to be a marker of visceral fat dysfunction (7). LAP can reflect the combined anatomic and physiologic changes associated with lipid over-accumulation in adults (8). Additionally, a Chinese visceral adiposity index (CVAI) is established for the evaluation of visceral fat dysfunction in Chinese populations, as the body fat distribution among various ethnicities is different and VAI has mainly been reported in Caucasians (9).

After menopause, adipose tissue is the major site in a woman's body to convert androgens to estrogens (10). Endogenous sex steroids mainly act on the reproduction system, but their roles in visceral obesity have not been completely revealed. As is known, postmenopausal women are more prone to be obese, mainly because the cessation of ovarian function after menopause results in withdrawal of ovarian sex steroids, estradiol (E_2_), and progesterone, which has been implicated in altered metabolism (11). Among them, E_2_ plays an important role in metabolic control and homeostasis, which has been reported to be associated with several metabolic disorders including abdominal obesity, insulin sensitivity, lipid transport, blood pressure, and inflammatory or prothrombotic states (12). Besides, in postmenopausal women, higher endogenous free testosterone (T), E_2_ and lower sex hormone-binding globulin (SHBG) were found to be associated with measures of adiposity (10, 13). Follicle-stimulating hormone (FSH) was also lower in women with obesity (14). Increased ratio of circulating levels of luteinizing hormone to FSH (LH/FSH ratio) was a common characteristic of women with polycystic ovary syndrome (PCOS) (15). It was also reported to be associated with insulin resistance and obesity, but mostly in the context of PCOS. To our knowledge, only one study examined the associations of LH/FSH ratio with overweight and obesity in the general postmenopausal women, which showed that the LH/FSH ratio was not significantly associated with obesity but did not explore the relationship between the LH/FSH ratio and visceral adipose function (15). Thus, little is known about the association of the LH/FSH ratio with visceral obesity after menopause.

Based on these, we hypothesized that the LH/FSH ratio was associated with visceral obesity in Chinese women older than 55 years. Accordingly, we performed a population-based observational investigation named Survey on Prevalence in East China for Metabolic Diseases and Risk Factors (SPECT-China) in 2014 and 2015. Using data from the study, we aimed to explore the association of the LH/FSH ratio with visceral obesity, including visceral adipose accumulation and function, in Chinese women over 55 years old who had a high probability of being postmenopausal.

## Materials and methods

### Study population

**Figure 1:**
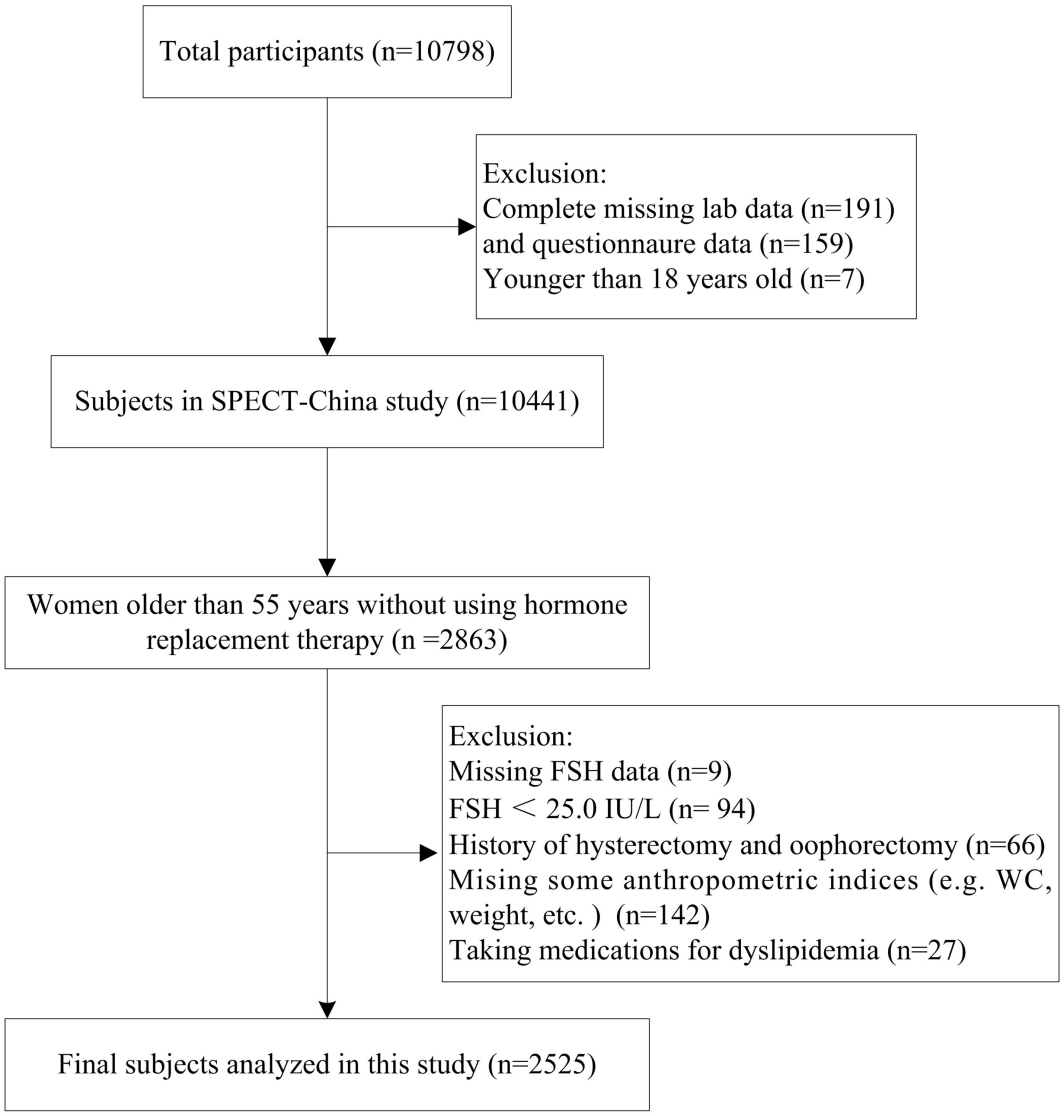
Flowchart of postmenopausal women selected from SPECT-China.

SPECT-China is a cross-sectional survey on the prevalence of metabolic diseases and risk factors in East China (ChiCTRECS-14005052, www.chictr.org.cn), which was conducted from February 2014 to December 2015. A stratified cluster sampling method was used. A total of 22 sites in Shanghai, Jiangxi Province, Zhejiang Province, Jiangsu Province and Anhui Province were selected. We invited Chinese adults aged 18 years and older who had lived in their current residence for 6 months or longer to participate in our study. Those with severe communication problems or acute illness and those who were unwilling to participate were excluded. A total of 10,798 residents participated in this investigation. After exclusion of participants who had completely missing laboratory results (*n* = 191), missing questionnaire data (*n* = 159) and were younger than 18 years old (*n* = 7), a total of 10,441 subjects were enrolled in SPECT-China study. Previous studies showed that women older than 55 had a high probability of being postmenopausal (16). In China, approximately 97% of women at the age of 55 are postmenopausal (17). There were 2,863 women over 55 years old who were not using hormone replacement therapy. Among them, those with missing values of FSH (*n* = 9), FSH < 25.0 IU/L (*n* = 94), and a history of hysterectomy and/or oophorectomy (*n* = 66) were excluded. Women missing some anthropometric data (e.g., WC, weight) (*n* = 142) or taking medications for dyslipidemia (*n* = 27) were also excluded. Finally, this study was based on a total number of 2,525 women who were older than 55 (Figure 1). The study protocol was approved by the Ethics Committee of Shanghai Ninth People's Hospital, Shanghai JiaoTong University School of Medicine. All procedures were in accordance with the ethical standards of the responsible committee on human experimentation (institutional and national) and with the Helsinki Declaration of 1975, as revised in 2008. All participants provided written informed consent before data collection.

### Measurements

In every site, the same trained staff group collected anthropometric data and completed the questionnaire including information on demographic characteristics, medical history and lifestyle risk factors. Current smoking was defined as having smoked at least 100 cigarettes in one's lifetime and currently smoking cigarettes (18). Body weight, height, waist circumference and blood pressure were measured with the use of standard methods as described previously (18). Body mass index (BMI) was calculated as weight in kilograms divided by height in meters squared. WHR was calculated as waist circumference divided by hip circumference. The VAI, CVAI, and LAP were calculated as follows (6, 9):

VAI = WC(cm)/[36.58 + 1.89 × BMI (kg/m^2^)] × [TG (mmol/L)/0.81] × [1.52/HDL (mmol/L)]
CVAI = −187.32 + 1.71 × age (y) + 4.32 × BMI (kg/m^2^) + 1.12 × WC (cm) + 39.76 × Log_10_TG (mmol/L) −11.66 × HDL (mmol/L)
LAP = [WC (cm) – 58] × TG (mmol/L)

Participants fasted for at least 8h before investigation. The blood samples for the plasma glucose test were collected into vacuum tubes with anticoagulant sodium fluoride and centrifuged on the spot within 1 h after collection. Blood samples were stored at –20°C when collected and were shipped by air in dry ice to a central laboratory within 2–4 h, which was certified by the College of American Pathologists. FPG, total cholesterol (TC), triglycerides (TG), high-density lipoprotein (LDL) and high-density lipoprotein (HDL) were measured by BECKMAN COULTER AU 680 (Brea, USA). Fasting insulin (FINS) was detected by chemiluminescence method (Abbott i2000 SR, USA). Insulin resistance was estimated by calculating the homeostatic model assessment (HOMA-IR) index: [fasting insulin (pmol/L) × fasting glucose (mg/dl)]/(22.5 × 6.965).

Total T, E_2_, FSH, and LH were measured by chemiluminescence (Siemens IMMULITE 2000, Munich, Germany). The minimal detectable limit for each hormone was as follows: 0.7 nmol/L (total T), 73.4 pmol/L (E_2_), and 0.1 IU/L (FSH and LH). The intra-assay variations for every hormone were calculated from three duplicate determinations in an assay. The inter-assay variations were calculated from results of 500 separate assays with duplicate samples in each assay. The inter-assay coefficients of variation were 6.6% (total T), 7.5% (E_2_), 4.5% (FSH), and 6.0% (LH). The intra-assay coefficients of variation were 5.7% (total T), 6.2% (E_2_), 3.8% (FSH), and 4.9% (LH). In this study, a part of total T (64.8%) and E_2_ (68.2%) was under the minimal detectable limit (19).

### Definition of variables and outcomes

Visceral obesity was defined as a waist circumference ≥80 cm in female (18). Hypertension was identified by SBP ≥140 mmHg, DBP ≥90 mmHg, or a self-reported previous diagnosis of hypertension by physicians. According to the modified National Cholesterol Education Program-Adult Treatment Panel III, dyslipidemia was defined as TC ≥6.22 mmol/L, TG ≥2.26 mmol/L, LDL ≥4.14 mmol/L or HDL <1.04 mmol/L, or treatment for hyperlipidemia by physicians (20).

### Statistical analysis

We performed survey analysis with IBM SPSS Statistics, Version 22 (IBM Corporation, Armonk, NY, USA). A two-sided *P*-value < 0.05 was considered significant. Continuous variables with a Gaussian distribution and categorical variables were expressed as the mean ± standard deviation (SD) and percentage (%), respectively. Continuous variables with a skewed distribution were presented as the median (interquartile range) and were log-transformed for analysis. To test the differences in characteristics between participants with and without visceral obesity and among different LH/FSH quartiles, Mann-Whitney *U-*test and Analysis of variance (ANOVA) were used for continuous variables with a Gaussian distribution, Student's t test and Kruskal-Wallis test for variables with a skewed distribution, and Pearson χ2 test for categorical variables. Spearman's correlation coefficient was employed to test the correlations between LH/FSH ratio and potential metabolic factors. The association of LH/FSH ratio (independent variable) with fatness indices including VAI, CVAI, and LAP (dependent variables) was assessed by linear regression. Model 1 controlled for age and smoking. Model 2 additionally controlled for HOMA-IR, hypertension and dyslipidemia. Model 3 additionally controlled for total T (proportion above vs. below detection limit) and E_2_ (proportion above vs. below detection limit). VAI, CVAI, and LAP were log-transformed because of their skewed distribution. Odds ratio (OR) and 95% confidence intervals (CIs) were calculated using binary logistic regression to determine the risk of visceral obesity for LH/FSH ratio. Model 1 adjusted for age and smoking. Model 2 additionally adjusted for HOMA-IR, hypertension and dyslipidemia. Model 3 additionally adjusted for total T (proportion above vs. below detection limit) and E_2_ (proportion above vs. below detection limit). In the linear regression and logistic regression, LH/FSH ratio was standardized to obtain its Zscore to explore the association of LH/FSH ratio with fatness indices and visceral obesity.

Sensitivity analyses were performed in these subjects by substituting BMI for WC in multivariable models. Additionally, a large investigation of 20,275 Chinese women found that at the age of 55, 97% of women were postmenopausal and at the age of 60, all women were postmenopausal (17). And, another study reported that FSH and E_2_ all achieved stability by 2 years after the final menstrual period (21). Based on theses, Chinese women at age of 65 were all postmenopausal and had no fluctuation of sex hormones. Thus, we also performed the regression analyses in women older than 65. Moreover, we categorized the subjects who reported to be postmenopausal according to the questionnaire and those who were older than 65 into the postmenopausal women category. Further sensitivity analyses were also conducted in these subjects.

## Results

### Characteristics of the study population

General demographic and laboratory characteristics of the participants were summarized in Table 1. This study recruited 2,525 postmenopausal women. Among them, a total of 1,462 (57.9%) had visceral obesity. Compared with subjects with non-visceral obesity, women with visceral obesity were older and had significantly greater fatness indices (WC, WHR, BMI, VAI, CVAI, LAP) and blood pressure as well as greater levels of FPG, FINS, HOMA-IR, TG, and LDL (all *P* < 0.001). Women with visceral obesity also had higher total T, LH/FSH ratio and lower levels of LH and FSH (all *P* < 0.001). Among them, the proportion of total T above the detection limit was 30.6% in non-visceral obese women and 38.5% in visceral obese women (*P* < 0.001). The proportion of E_2_ above the detection limit was 28.4% in non-visceral obese women and 34.2% in visceral obese women (*P* < 0.01). In contrast, the level of HDL was significantly lower in women with visceral obesity.

**Table 1:** General characteristics of the subjects categorized by waist circumference.

	**Non-visceral obesity**	**Visceral obesity**	**Relative change percentage (%)**	***P***
*N*	1,063	1,462		
Age (year)	63.31 ± 6.61	65.02 ± 6.5	2.7	< 0.001
**METABOLIC FACTORS**				
Waist circumference (cm)	72.57 ± 4.99	88.06 ± 6.84	21.3	< 0.001
Waist-hip ratio	0.82 ± 0.05	0.91 ± 0.12	11.0	< 0.001
Body mass index (kg/m^2^)	22.55 ± 2.73	26.69 ± 3.21	18.4	< 0.001
Visceral adiposity index	1.40 (1.19)	2.20 (1.61)	48.4	< 0.001
Chinese visceral adiposity index	84.28 ± 22.78	127.41 ± 24.62	51.2	< 0.001
Lipid accumulation product	18.98 (17.08)	47.16 (34.12)	151	< 0.001
Systolic pressure (mmHg)	135.13 ± 21.70	144.11 ± 21.18	6.6	< 0.001
Diastolic pressure (mmHg)	77.39 ± 12.28	82.04 ± 12.49	6.0	< 0.001
Fasting glucose (mmol/L)	5.66 ± 1.19	6.02 ± 1.74	6.4	< 0.001
Fasting insulin (pmol/L)	29.10 (16.60)	41.40 (27.45)	44.7	< 0.001
HOMA-IR	1.03 (0.64)	1.51 (1.16)	55.6	< 0.001
TG (mmol/L)	1.24 (0.79)	1.58 (0.97)	25.5	< 0.001
TC (mmol/L)	5.44 ± 1.08	5.51 ± 1.21	1.3	0.161
LDL (mmol/L)	3.20 ± 0.80	3.36 ± 0.82	5.0	< 0.001
HDL (mmol/L)	1.56 ± 0.34	1.42 ± 0.30	−9.0	< 0.001
**SEX HORMONES**				
Total T (proportion above vs. below detection limit, %)	30.6 vs. 69.4	38.5 vs. 61.5		< 0.001
E_2_ (proportion above vs. below detection limit, %)	28.4 vs. 71.6	34.2 vs. 65.8		< 0.01
LH (IU/L)	25.80 (14.30)	22.70 (12.10)	−11.2	< 0.001
FSH (IU/L)	67.50 (32.10)	56.20 (27.35)	−15.5	< 0.001
LH/FSH ratio	0.38 (0.15)	0.40 (0.15)	5.0	< 0.001
Current smoker (%)	3.0	3.9		0.269

*Data are summarized as the mean ± standard deviation or median (interquartile range) for normally or non-normally distributed continuous variables and as number with proportion for categorical variables. The Mann-Whitney U or Student's T-test was used for continuous variables, and the Pearson χ2 test was used for dichotomous variables*.

Characteristics of subjects by LH/FSH quartiles are shown in Table 2. The quartile ranges of LH/FSH in subjects were ≤ 0.32, 0.33–0.39, 0.40–0.47, and ≥0.48. Compared with women in the lowest quartile, women in the highest quartile were younger but had greater fatness indices, blood pressure, FINS, TG and LDL (all *P* < 0.05). These women also had higher total T and LH but significantly lower FSH (all *P* < 0.05). Among them, the proportion of total T above the detection limit was 28.5, 33.2, 36.7, and 42.6% respectively from the lowest to the highest quartile (*P* < 0.001). The proportion of E_2_ above the detection limit was 32.6, 32.6, 28.5, and 33.3% respectively in the four quartiles (*P* > 0.05).

**Table 2:** Characteristics of the subjects according to LH/FSH quartiles.

**LH/FSH ratio**	**Q1**	**Q2**	**Q3**	**Q4**	***P* for trend**
	≤0.32	0.33–0.39	0.40–0.47	≥0.48	
*N*	641	645	624	615	
Age (year)	65.31 ± 6.96	64.53 ± 6.76	63.87 ± 6.32	63.42 ± 6.16	< 0.001
**METABOLIC FACTORS**					
Waist circumference (cm)	80.33 ± 9.89	80.85 ± 9.43	81.76 ± 9.48	83.30 ± 10.17	< 0.001
Waist-hip ratio	0.87 ± 0.08	0.87 ± 0.08	0.87 ± 0.17	0.88 ± 0.08	0.05
Body mass index (kg/m^2^)	24.43 ± 3.57	24.68 ± 3.59	25.07 ± 3.47	25.64 ± 3.84	< 0.001
Visceral adiposity index	1.61 (1.58)	1.83 (1.52)	1.93 (1.50)	1.97 (1.63)	< 0.001
Chinese visceral adiposity index	105.87 ± 32.15	107.26 ± 31.78	109.98 ± 30.91	114.13 ± 32.54	< 0.001
Lipid accumulation product	30.38 (31.44)	33.82 (32.27)	34.12 (33.70)	37.41 (36.66)	< 0.001
Systolic pressure (mmHg)	138.93 ± 20.81	138.32 ± 21.50	141.73 ± 22.20	142.41 ± 22.67	< 0.01
Diastolic pressure (mmHg)	78.89 ± 11.90	78.89 ± 12.50	80.97 ± 13.10	81.63 ± 12.73	< 0.001
Fasting glucose (mmol/L)	5.86 ± 1.31	5.92 ± 1.78	5.80 ± 1.41	5.89 ± 1.62	0.53
Fasting insulin (pmol/L)	32.50 (21.70)	33.90 (25.30)	36.60 (22.90)	37.00 (26.60)	< 0.05
HOMA-IR	1.18 (0.88)	1.23 (0.98)	1.33 (0.96)	1.33 (1.11)	0.06
TG (mmol/L)	1.32 (0.91)	1.48 (0.96)	1.48 (0.94)	1.48 (0.95)	< 0.01
TC (mmol/L)	5.49 ± 1.11	5.49 ± 1.04	5.46 ± 1.31	5.48 ± 1.14	0.86
LDL (mmol/L)	3.18 ± 0.78	3.25 ± 0.79	3.33 ± 0.82	3.42 ± 0.87	< 0.001
HDL (mmol/L)	1.54 ± 0.34	1.51 ± 0.33	1.44 ± 0.31	1.41 ± 0.31	< 0.001
**SEX HORMONES**					
Total T (proportion above vs. below detection limit, %)	28.5 vs. 71.5	33.2 vs. 66.8	36.7 vs. 63.3	42.6 vs. 57.4	< 0.001
E_2_ (proportion above vs. below detection limit, %)	32.6 vs.67.4	32.6 vs. 67.4	28.5 vs. 71.5	33.3 vs. 66.7	0.249
LH (IU/L)	18.5 (10.3)	23.1 (10.6)	25.3 (12.9)	31.1 (14.4)	< 0.001
FSH (IU/L)	66.4 (33.0)	63.4 (29.8)	59.4 (29.7)	53.8 (25.5)	< 0.001
Current smoker (%)	4.7	3.1	3.1	3.2	0.33

*Data are summarized as the mean ± standard deviation or median (interquartile range) for normally or non-normally distributed continuous variables and as the number with proportion for categorical variables. ANOVA and Kruskal-Wallis test were used for continuous variables, and the Pearson χ2 test was used for dichotomous variables*.

### Association of LH/FSH ratio with visceral adiposity indicators

Table 3 summarized the results of the linear regression models studying the association of LH/FSH ratio with visceral obesity indicators. In the base model (Table 3, model 1), higher LH/FSH ratio was associated with higher levels of all the fatness indices [B (95% CI): Log VAI 0.094 (0.061–0.127), Log CVAI 0.062 (0.046–0.078), Log LAP 0.130 (0.088–0.172), all *P* < 0.001]. Further adjustment for HOMA-IR, hypertension and dyslipidemia attenuated the association [B (95% CI): Log VAI 0.062 (0.032–0.092), Log CVAI 0.050 (0.034–0.066), Log LAP 0.110 (0.070–0.149), all *P* < 0.001] but enhanced *R*^2^ (Table 3, model 2). After further controlling for total T (proportion above vs. below detection limit) and E_2_ (proportion above vs. below detection limit), this association largely weakened [B (95% CI): Log VAI 0.060 (0.030–0.090), Log CVAI 0.045 (0.029–0.061), Log LAP 0.103 (0.063–0.142), all *P* < 0.001], but *R*^2^ was elevated (Table 3, model 3). In all these models, the positive association of LH/FSH ratio with these adiposity indicators was always statistically significant.

**Table 3:** Association of LH/FSH ratio with visceral adiposity indicators: linear regression.

**Dependent variables**	**β**	**B (Per-SD increase)**	**95% CI**	***P***	***R*^2^**
Log VAI (model 1)	0.113	0.094	0.061–0.127	< 0.001	0.014
Log VAI (model 2)	0.073	0.062	0.032–0.092	< 0.001	0.278
Log VAI (model 3)	0.070	0.060	0.030–0.090	< 0.001	0.280
Log CVAI (model 1)	0.141	0.062	0.046–0.078	< 0.001	0.169
Log CVAI (model 2)	0.112	0.050	0.034–0.066	< 0.001	0.283
Log CVAI (model 3)	0.101	0.045	0.029–0.061	< 0.001	0.293
Log LAP (model 1)	0.122	0.130	0.088–0.172	< 0.001	0.022
Log LAP (model 2)	0.101	0.110	0.070–0.149	< 0.001	0.248
Log LAP (model 3)	0.094	0.103	0.063–0.142	< 0.001	0.251

*Model 1 adjusted for age and smoking*.

*Model 2 adjusted for age, smoking, HOMA-IR, hypertension and dyslipidemia*.

*Model 3 adjusted for age, smoking, HOMA-IR, hypertension, dyslipidemia, total testosterone (proportion above vs. below detection limit) and estradiol (proportion above vs. below detection limit)*.

### Association of LH/FSH ratio with visceral obesity

Binary logistic regression analyses (Table 4) showed that the risk of visceral obesity increased with increasing LH/FSH ratio (*P* < 0.001 in every model). In the model adjusted for age and smoking (Table 4, model 1), the OR of visceral obesity was 1.99 (95% CI 1.52, 2.59; *P* < 0.001). After additional adjustments for HOMA-IR, hypertension and dyslipidemia (Table 4, model 2), the OR clearly decreased to 1.89 (95%CI 1.42, 2.53), but there was still statistical significance (*P* < 0.001). Further adjustment for total T (proportion above vs. below detection limit) and E_2_ (proportion above vs. below detection limit) did not greatly weaken the association of LH/FSH ratio with visceral obesity (Table 4, model 3). The OR of visceral obesity in model 3 was 1.83 (95% CI 1.37, 2.45) (*P* < 0.001).

**Table 4:** Association of LH/FSH ratio with visceral obesity: logistic regression.

	**Model 1 (Per-SD increase)**	**Model 2 (Per-SD increase)**	**Model 3 (Per-SD increase)**
Zscore(LH/FSH ratio)	1.99 (1.52, 2.59)^***^	1.89 (1.42, 2.53)^***^	1.83 (1.37, 2.45)^***^
Age	1.04 (1.03, 1.06)^***^	1.03 (1.02, 1.05)^***^	1.03 (1.02, 1.05)^***^
Smoking	0.90 (0.57, 1.41)	0.83 (0.52, 1.32)	0.82 (0.51, 1.32)
HOMA-IR		1.42 (1.29, 1.57)^***^	1.41 (1.28, 1.55)^***^
Hypertension		1.74 (1.45, 2.09)^***^	1.72 (1.43, 2.06)^***^
Dyslipidemia		1.33 (1.11, 1.60)^**^	1.35 (1.12, 1.62)^**^
Total T (proportion above vs. below detection limit)			1.27 (1.05, 1.52)^*^
E_2_ (proportion above vs. below detection limit)			0.88 (1.07, 1.30)

*Data are expressed as the odds ratio (95% confidence interval)*.

*Model 1 controlled for age and smoking*.

*Model 2 additionally controlled for HOMA-IR, hypertension and dyslipidemia*.

*Model 3 additionally controlled for total T (proportion above vs. below detection limit) and E_2_ (proportion above vs. below detection limit)*.

* P < 0.05,

** P < 0.01,

*** *P < 0.001*.

### Sensitivity analysis

In the sensitivity analysis, using an obesity category by BMI instead of WC in the three models did not change the observed association (Table 5). We also increased the cutoff age of menopause to 65 years old (*n* = 1,063), and a significant association still existed in the three different models (*P* for trend < 0.01). Moreover, we categorized the subjects who reported to be postmenopausal according to the questionnaire (part of them had these data) and those who were older than 65 into the postmenopausal women category (*n* = 1,625). In these subjects, LH/FSH ratio was still positively associated with visceral obesity, even in the fully adjusted model (*P* for trend < 0.01) (Table 6).

**Table 5:** Association of LH/FSH ratio with obesity categorized by BMI: logistic regression.

	**Model 1 (Per-SD increase)**	**Model 2 (Per-SD increase)**	**Model 3 (Per-SD increase)**
BMI < 25	Ref.	Ref.	Ref.
BMI 25–29.9	1.62 (1.24, 2.12)^***^	1.46 (1.09, 1.95)^*^	1.32 (0.99, 1.77)
BMI ≥ 30	2.39 (1.55, 3.68)^***^	2.36 (1.50, 3.71)^***^	2.07 (1.31, 3.28)^**^

*Data are expressed as the odds ratio (95% confidence interval)*.

*Model 1 controlled for age and smoking*.

*Model 2 additionally controlled for HOMA-IR, hypertension and dyslipidemia*.

*Model 3 additionally controlled for total testosterone (proportion above vs. below detection limit) and estradiol (proportion above vs. below detection limit)*.

* P < 0.05,

** P < 0.01,

*** *P < 0.001*.

**Table 6:** Association of LH/FSH ratio with visceral obesity.

	**Postmenopausal women (*****n*** = **1625)**		**Women** ≥ **65 years old (*****n*** = **1063)**	
**Linear regression**	**B (95% CI) Per-SD increase**	***R*^2^**	**B (95% CI) Per-SD increase**	***R*^2^**
Log VAI (model 1)	0.101 (0.061–0.141)^***^	0.016	0.128 (0.076-0.180)^***^	0.024
Log VAI (model 2)	0.068 (0.031–0.105)^***^	0.279	0.065 (0.018–0.112)^**^	0.300
Log VAI (model 3)	0.064 (0.027–0.101)^**^	0.282	0.060 (0.013–0.108)^*^	0.302
Log CVAI (model 1)	0.063 (0.045–0.081)^***^	0.141	0.074 (0.051–0.096)^***^	0.081
Log CVAI (model 2)	0.054 (0.036–0.073)^***^	0.257	0.055 (0.033–0.078)^***^	0.229
Log CVAI (model 3)	0.051 (0.032–0.070)^***^	0.268	0.049 (0.027–0.072)^***^	0.246
Log LAP (model 1)	0.131 (0.080–0.182)^***^	0.017	0.180 (0.115–0.244)^***^	0.030
Log LAP (model 2)	0.116 (0.068–0.165)^***^	0.241	0.123 (0.061–0.185)^***^	0.263
Log LAP (model 3)	0.112 (0.063–0.160)^***^	0.245	0.115 (0.053–0.177)^***^	0.268
**Logistic regression**	**OR (95% CI) Per-SD increase**		**OR (95% CI) Per-SD increase**	
Visceral obesity (Model 1)	1.88 (1.35, 2.62)^***^		3.21 (2.01, 5.12)^***^	
Visceral obesity (Model 2)	1.85 (1.28, 2.68)^**^		2.52 (1.52, 4.18)^***^	
Visceral obesity (Model 3)	1.86 (1.28, 2.70)^**^		2.49 (1.50, 4.14)^***^	

*Model 1 adjusted for age and smoking*.

*Model 2 adjusted for age, smoking, HOMA-IR, hypertension and dyslipidemia*.

*Model 3 adjusted for age, smoking, HOMA-IR, hypertension, dyslipidemia, total testosterone (proportion above vs. below detection limit) and estradiol (proportion above vs. below detection limit)*.

* P < 0.01,

** P < 0.001,

*** *P < 0.001*.

## Discussion

In this study, we found that a higher LH/FSH ratio was significantly associated with visceral adipose over-accumulation and dysfunction defined by LAP, VAI and CVAI and with a higher risk of visceral obesity in Chinese women over 55 years old who had a high probability of being postmenopausal. This study was a population-based investigation with a large sample that aimed to detect the association between LH/FSH ratio and visceral obesity including both adipose distribution and function.

Menopause, best defined as the absence of menses for 12 consecutive months, is the most reliable indicator of the postmenopausal state and is frequently associated with an increase in visceral fat. During menopausal transitions, ovarian hormones declined dramatically, which would influence the accumulation, regional distribution, function and metabolism of visceral adipose tissue and increase the risk of cardiovascular disease (CVD) (2). Menopause-related increase in central or abdominal obesity, independent of age and total body fat mass, had been documented in many studies across multiple cultures (22, 23). Animal studies also indicate that estrogen depletion favored central abdominal fat accumulation (24). In contrast, postmenopausal visceral obesity could also influence changes in sex hormones and their binding protein concentrations (10, 25). After menopause, the hypothalamic-pituitary-gonadal axis is known to not function comparably in postmenopausal women because of the cessation of estradiol production by the granulosa cells. Alternatively, adipose tissue serves as the major site of peripheral aromatization of androgens to estrogens (2, 22). Thus, although menopause was characterized by decreased E_2_ levels and elevated FSH (26), many studies (13, 26) found higher E_2_ and lower LH and FSH levels in obese individuals. In our study, increased E_2_ levels and decreased FSH and LH levels were also found in obese women over 55 years old. Furthermore, adipose tissue expresses receptors for sex steroid hormones, including estrogen receptor, progesterone receptor and androgen receptor. Studies in receptor knockout animal models demonstrated that deficiency in estrogen and/or estrogen receptor produced weight gain, increased visceral adiposity, and impaired glucose/insulin tolerance (2, 24). However, we found few studies were conducted to explore the relationship between LH/FSH ratio and visceral obesity in postmenopausal women outside the context of PCOS.

High LH/FSH ratio is often thought to be one pathway leading to hyperandrogenism in PCOS women, although the findings remain inconclusive. The association of LH/FSH ratio with obesity (overall and visceral) (15), insulin resistance (15), hyperglycemia (27), and even chronic inflammation (28) has mostly been explored in the context of PCOS women. To our knowledge, until now, only one study explored the association of LH/FSH ratio with visceral obesity in postmenopausal women, and that study showed no statistical differences and did not explore the relationship between LH/FSH ratio and visceral adipose function (15). As is known, the reason why visceral obesity is more likely to lead to metabolic disorders may be attributed to not only visceral adipose accumulation but also visceral adipose dysfunction. Visceral adipose dysfunction, manifesting as abnormal fatty acid metabolism, increased oxidative stress, endothelial dysfunction, and excessive production of adipokines has been proposed in the pathogenesis of MetS (2). However, neither WC nor WHR can reflect visceral adipose function, and they cannot even accurately estimate visceral adipose distribution. Thus, we used LAP, VAI, and CVAI to evaluate visceral adipose accumulation and function. VAI was shown to be strongly associated with visceral adipose tissue measured with magnetic resonance imaging (MRI) (29) and would be an easy tool for clearly reflecting adipose tissue dysfunction among the most common indices of adiposity assessment including BMI, WC, WHR and adipocytokines (30). CVAI was demonstrated to be a reliable and applicable index for the evaluation of visceral fat dysfunction in Chinese populations (9). LAP is an indicator used to describe the extent to which WC and TG increased. Ectopic lipid deposition was difficult to quantify directly, but an increased LAP value might indicate that various tissues or organs had become more vulnerable to injury from lipid over-accumulation. Our study found that LH/FSH ratio was positively associated with VAI, CVAI and LAP, even after controlling for possible confounders. In addition, with increases in LH/FSH ratio, the values of VAI, CVAI and LAP increased gradually. Beydoun et al. reported that LH/FSH ratio was not significantly associated with obesity, categorized by BMI or WC, in the general postmenopausal women. Additionally, the authors did not explore the relationship between LH/FSH ratio and visceral adipose function, which could be reflected by VAI. In our study, we found that LH/FSH ratio was not only positively associated with visceral obesity categorized by WC but also positively associated with visceral adipose dysfunction. The inconsistency between the results found by Beydoun et al. and our own may be attributed to the different ethnicities included, which needs further study.

The mechanisms between sex hormones and visceral obesity remain not completely revealed. Many studies had documented that estrogen deficiency promoted central fat accumulation through the following possible mechanisms: adipocyte hypertrophy, adipose tissue oxidative stress and inflammation (31), increasing the uptake of lipids from circulation (32) and reducing energy expenditure (24). Some studies also proved that deleterious changes in inflammatory markers and adipokines correlated strongly with increased visceral adiposity at menopause (33). Catherine Kim et al. (14) thought that decreased FSH levels in postmenopausal overweight and obese women resulted from changes in E_2_ concentrations or other adiposity-related markers. Sowers et al. (34) even reported that genetic factors might link menopausal and obesity. Estrogen receptor (ER) and progesterone receptor (PR) had also been shown to influence metabolism and inflammatory responses (11). ERα and ERβ were reported to be involved in blood glucose and lipid homeostasis (35). ERα knockout (ERαKO) mice as well as aromatase knockout (ArKO) mice were obese and insulin resistant (36). However, the exact pathophysiology of the relationship between LH/FSH ratio and visceral obesity has not been well determined in postmenopausal women. Also, it may be attributed to the changes in E_2_ or progesterone concentrations or other adiposity-related markers., which merits further investigations.

Our study had some strengths. First, the novel findings; it is a study with a large population-based sample to detect the association of LH/FSH ratio with both visceral adipose over-accumulation and dysfunction. Second, the study has strong quality control, as the anthropometric measurements and questionnaires were completed by the same trained research group and as all biomedical measurements were performed in one laboratory certified by the College of American Pathologists. Third, the study data are from the SPECT-China study, which was performed in a general population, as opposed to a clinic-based population, so the results may be more reflective. However, there are also some limitations. First, as this is a cross-sectional study, we cannot draw a causal relationship between LH/FSH ratio and visceral obesity. Second, computed tomography (CT) and MRI are currently considered the most accurate direct measurements of body fat distribution (4). However, because these two imaging techniques are expensive and require specialized equipment, they are not appropriate for large epidemiological studies. Additionally, VAI is strongly associated with visceral adipose tissue measured with MRI (29), and thus VAI could be used as a useful tool for reflecting visceral adipose distribution and dysfunction. Third, this study recruited primarily Han Chinese people. Whether the data could be generalizable to other ethnic or racial groups warrants further investigation. Another limitation is that a large part of total T (64.8%) and E_2_ (68.2%) concentrations was under the minimal detectable limit. In order to avoid the interference of total T and E_2_ on the results, we controlled for total T (proportion above vs. below detection limit) and E_2_ (proportion above vs. below detection limit) in the fully adjusted model when conducting linear regression and logistic regression analyses. The results also showed LH/FSH ratio was positively associated with visceral obesity in women over 55 years old, which means these results were credible and meaningful. Finally, in our study, unfortunately, not all women had the data of the menopause age in the questionnaire and women older than 55 were considered to have a high probability of being postmenopausal. Previous studies reported that the overall median age at natural menopause was 50 years old and 97% of women were postmenopausal at the age of 55, and at the age of 60, all women were postmenopausal in China (17). This age cutoff has been used in many previous studies (14, 16, 19, 37). Besides, FSH and E_2_ achieved stability by 2 years after the final menstrual period (21). Thus, to avoid the influence of fluctuation of sex hormones, we increased the cutoff age of menopause to 65 in the sensitivity analysis. A significant association between LH/FSH ratio and visceral obesity still existed. Moreover, we further performed the regression analyses in women who reported to be postmenopausal according to the questionnaire and those who were older than 65. The observed association was also not changed. Based on these, we do not think that this assumption would seriously bias this study.

## Conclusions

An increased LH/FSH ratio was significantly associated with visceral adipose over-accumulation and dysfunction in Chinese women older than 55 who had a high probability of being postmenopausal. Therefore, the LH/FSH ratio may be an early marker for metabolic disorders in Chinese women over 55 years old, which warrants further investigation. This relationship should be further confirmed in the context of large prospective cohort studies of pre-, peri- and post-menopausal women. Further investigations are also needed to explore the possible mechanisms behind this association.

## Author contributions

YL designed the study; LZ, CZ, YC, CC, JC, FX, and NW participated in acquisition of data; YL and NW evaluated the literature; LZ undertook the statistical analysis and wrote the first draft of the manuscript. YL and NW edited and revised the manuscript. All authors read and approved the final manuscript for publication.

### Conflict of interest statement

The authors declare that the research was conducted in the absence of any commercial or financial relationships that could be construed as a potential conflict of interest.

## Abbreviations

WC: waist circumference
BMI: body mass index
WHR: waist-to-hip ratio
SBP: systolic blood pressure
DBP: diastolic blood pressure
FPG: fasting plasma glucose
FINS: fasting insulin
HOMA-IR: insulin resistance index
TG: triglycerides
TC: total cholesterol
LDL: low-density lipoprotein
HDL: high-density lipoprotein
VAI: visceral adiposity index
CVAI: Chinese visceral adiposity index
LAP: lipid accumulation product
T: testosterone
E_2_: estradiol
LH: luteinizing hormone
FSH: follicle-stimulating hormone.
